# Integrating Health Inequality Monitoring and Equity-Focused Policy Analysis for Immunization: A Conceptual Framework for Translating Data into Equity-Oriented Action

**DOI:** 10.3390/vaccines14030219

**Published:** 2026-02-27

**Authors:** Anelisa Jaca, Lindi Mathebula

**Affiliations:** 1 Cochrane Centre South Africa, South African Medical Research Council, Cape Town 8000, South Africa; 2 Department of Public Health, Nelson Mandela University, Gqeberha 6031, South Africa

**Keywords:** health inequality monitoring, equity, zero-dose children, Immunization Agenda 2030, data-to-policy

## Abstract

Background: Although global immunization coverage has increased substantially, significant inequalities persist. Despite the availability of disaggregated data, inequality evidence is rarely translated into equity-oriented policy action. This gap reflects the weak integration between Health Inequality Monitoring (HIM) and Health Policy Analysis (HPA), which limits the use of data in immunization decision-making. Objective: This paper presents a conceptual framework and accompanying protocol that integrates HIM with equity-focused HPA. The framework is supported by public health informatics to enable the systematic translation of immunization inequality evidence into actionable policy. Methods: The framework adopts a mixed-methods approach aligned with World Health Organization (WHO) standards. Quantitative Health Inequality Monitoring (HIM) is used to measure absolute and relative inequalities in key immunization indicators, including DTP3 coverage and zero-dose prevalence, across socio-economic and geographic dimensions. Qualitative policy analysis draws on the Health Policy Analysis (HPA) triangle and the Health Equity Assessment Policy (HEAP) framework to examine the actors, policy content, processes, and contextual factors underlying these disparities. Public health informatics connects these components by enabling data interoperability, real-time visualization, and the routine embedding of inequality evidence within digital health information systems. Results: The resulting informatics-enabled data-to-policy pathway formalizes the link between measurement and policy action. Key outputs include automated equity dashboards and stakeholder-informed policy briefs, which are designed to support evidence-based prioritization, planning, and resource allocation. Conclusions: By integrating health inequality monitoring, policy analysis, and public health informatics, this framework directly addresses the ‘know–do’ gap in immunization equity. It offers a scalable and practical model for operationalizing the Immunization Agenda 2030, particularly in resource-constrained health systems.

## 1. Introduction

Immunization is an essential pillar of global public health and it is estimated that it will save 5.1 million lives by 2030 through preventing vaccine-preventable diseases [1]. Its most profound impact occurs in low- and middle-income countries (LMICs). Over the past two decades, global vaccination coverage has improved significantly, driven by major international initiatives aimed at reducing immunization inequities, including the Sustainable Development Goals (SDGs), the establishment of Gavi, the Vaccine Alliance, and the Global Vaccine Action Plan 2011–2020 (GVAP) [2]. However, progress has been uneven and inequitable. The coverage of three doses of the diphtheria–tetanus–pertussis vaccine (DTP3), a key proxy indicator for immunization coverage, has stalled at approximately 85% in 2024 [3]. Only 57% of countries have reached the critical target of at least 90% coverage [3]. Alarmingly, an estimated 14.3 million infants did not receive even a first dose of DTP in 2024, highlighting the persistent gaps in access to routine immunization services that disproportionately affect disadvantaged and underserved populations [3]. Although the Immunization Agenda 2030 (IA2030) emphasizes equity and the pledge to “leave no one behind,” significant disparities in coverage and outcomes will continue to exist unless immediate action is taken [2].

Health Inequality Monitoring (HIM) plays a decisive role in advancing immunization equity by measuring health disparities among population subgroups [4]. It is vital to recognize that immunization equity stipulates that everyone must “achieve their full health potential regardless of demographic, socio-economic, or geographic factors”. Measuring immunization inequalities is crucial for identifying population groups with limited access to vaccines and for providing the evidence necessary to guide effective and equitable policies, programs, and practices [5]. The Health Policy Analysis (HPA) triangle complements HIM by systematically evaluating the policy content, implementation, and effects of health policies on public health outcomes [6]. HIM equips decision-makers with the tools to interpret inequality patterns, understand how immunization policies affect various population groups, and prioritize resource allocation effectively [7].

The HIM methodology quantifies inequalities in immunization coverage, access, and outcomes by leveraging disaggregated data on crucial immunization health indicators (e.g., DTP3 coverage) and dimensions of inequality (e.g., socio-economic status, education, place of residence, and geographic areas) [8]. Monitoring these inequalities is essential for identifying groups that are consistently being overlooked, allowing for the formulation of precise policies and programs aimed at closing those gaps [9]. By making disparities visible and comparable over time, HIM generates vital evidence that identifies underserved populations and tracks progress toward equity goals [5]. Despite advancements in both HIM and HPA, the integration of inequality data into health policy remains insufficient. This disconnect inhibits the ability to leverage evidence-based insights effectively for shaping immunization policy and strategic decision-making. Aligning HIM with HPA is not just beneficial but imperative for transforming evidence into meaningful action, ensuring that immunization policies and programs are fundamentally anchored in equity.

Public Health Informatics (PHI) serves as a critical bridge connecting HIM and HPA, empowering the translation of evidence on inequality into actionable, equity-focused policies and programs. PHI encompasses the necessary tools, systems, and processes for the collection, integration, management, and analysis of large-scale health data, including immunization coverage disaggregated by key equity factors [10]. By linking high-quality data from HIM with analytical frameworks from HPA, PHI facilitates the timely identification of populations missed by vaccination services, evaluates policy impacts on different subgroups, and promotes evidence-informed decision-making. Furthermore, PHI enhances the visualization, dissemination, and routine application of inequality data by policymakers and program managers, ensuring that immunization strategies are targeted, equitable, and responsive to real-world needs [11]. In this way, PHI not only strengthens the technical capacity to monitor inequalities but also drives the evidence-to-policy translation, decisively addressing one of the most significant barriers to achieving equitable immunization coverage globally [12].

The primary challenge in tackling immunization inequalities does not stem from a lack of data, but from the inadequate and inconsistent translation of inequality evidence into policy-relevant action [13]. While substantial disaggregated data on immunization coverage exists, weaknesses in inequality monitoring, such as gaps in data use, analytical capacity, interpretation, and integration into routine policy and planning, prevent this evidence from being systematically applied to inform critical decisions [4]. Consequently, health systems often fail to identify populations that remain persistently under-vaccinated and do not prioritize essential equity-focused interventions [14]. Immediate and concerted action is necessary to rectify these shortcomings and ensure that all populations receive the immunization services they need.

## 2. Aim and Objectives

### 2.1. Aim

To outline a conceptual framework for integrating HIM and equity-focused HPA, supported by public health informatics, to enhance decision-making in equity-oriented immunization policies and programs.

### 2.2. Objectives

1. Apply standardized HIM methods to immunization data.
2. Quantify immunization inequalities across key dimensions and health indicators.
3. Integrate findings on inequality into equity-focused policy analysis.
4. Develop informatics-enabled decision-support tools.
5. Strengthen the translation of data into policy for immunization equity.

## 3. Methodology

This framework presents a mixed-methods design that combines HIM, HPA, and PHI.

### 3.1. Ethics Considerations

In the first phase, the study will utilize publicly available secondary data devoid of individual identifiers. When necessary, quantitative and policy data will be anonymized and managed in accordance with data protection standards. For the second phase, which involves stakeholder engagement through interviews and focus group discussions (FGDs), we will seek ethical approval from the relevant ethics committees as needed.

### 3.2. Health Inequality Monitoring

**Figure 1:**
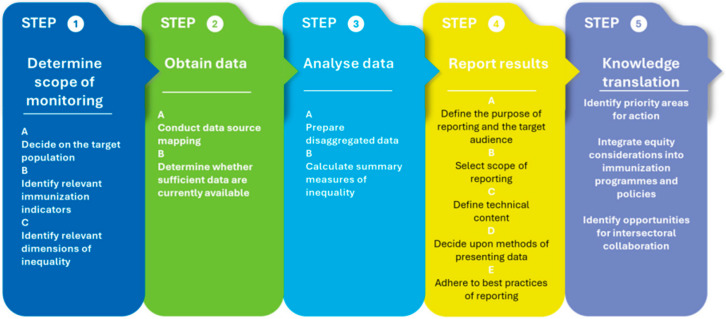
The Five Stages of the Health Inequality Monitoring Method. Adapted from the World Health Organization. Monitoring, Evaluation, and Review of National Health Strategies: A Country-Led Platform for Information and Accountability. WHO: Geneva, Switzerland, 2024.

We will use the World Health Organization (WHO)’s HIM methodology to quantify inequalities, aiming to improve the uptake and implementation of inequality monitoring practices in immunization [8]. We will follow the five steps of HIM, as detailed below (Figure 1).

#### 3.2.1. Determining the Scope of Monitoring

This initial step involves deciding on the target population, identifying relevant immunization indicators, and determining the dimensions of inequality to monitor. It includes mapping and engaging stakeholders to identify priority research areas and the necessary information. We will also map and review existing immunization policies and programs to pinpoint target populations, health indicators, and prioritized dimensions of inequalities [8].

- Target population

Depending on the scope of our research, we will identify the priority target populations (e.g., by country, province, district, etc.) for monitoring inequalities.

- Immunization health indicators

**Figure 2:**
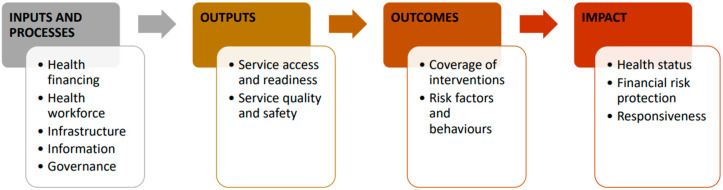
The Monitoring, Evaluation, and Review Framework. Adapted from the World Health Organization. Monitoring, Evaluation, and Review of National Health Strategies: A Country-Led Platform for Information and Accountability. WHO: Geneva, Switzerland, 2024.

In collaboration with relevant stakeholders, we will utilize a monitoring, evaluation, and review framework to identify pertinent health indicators for tracking immunization inequalities (Figure 2). This framework comprises four domains: (i) inputs and processes, (ii) outputs, (iii) outcomes, and (iv) impact. Each domain includes specific health indicators. The input and processes domain includes indicators related to health financing, workforce, infrastructure, information, and governance. The output domain encompasses service access, readiness, quality, and safety indicators. The outcome domain covers intervention coverage, risk factors, and behaviors, while the impact domain consists of health status, financial risk protection, and responsiveness indicators. For our study, we will focus on health indicators from the outputs and outcomes domains, specifically service access and intervention coverage.

- Dimensions of inequality

We will identify and select relevant dimensions of inequality in immunization, taking the target population into account. Commonly considered dimensions of inequality include household economic status, parental education level (especially maternal), place of residence, sex, mother’s age, and other country- or context-specific factors such as religion, tribe, and ethnicity. We will also evaluate any overlaps among the dimensions of inequality and consider whether double disaggregation is necessary. For each selected dimension, we will establish measurement criteria.

#### 3.2.2. Obtain Data

To measure health inequalities in immunization, two types of data are required, i.e., health indicators and inequality dimensions [8].

- Data source mapping

We will conduct data source mapping to identify which sources contain data about immunization health indicators and dimensions of inequality. The commonly used data sources for immunization coverage indicators include household surveys, such as the Demographic and Health Surveys (DHS) and the Multiple Indicator Cluster Surveys (MICS), as well as administrative data that consist of records kept by health facilities or the health system.

**Figure 3:**
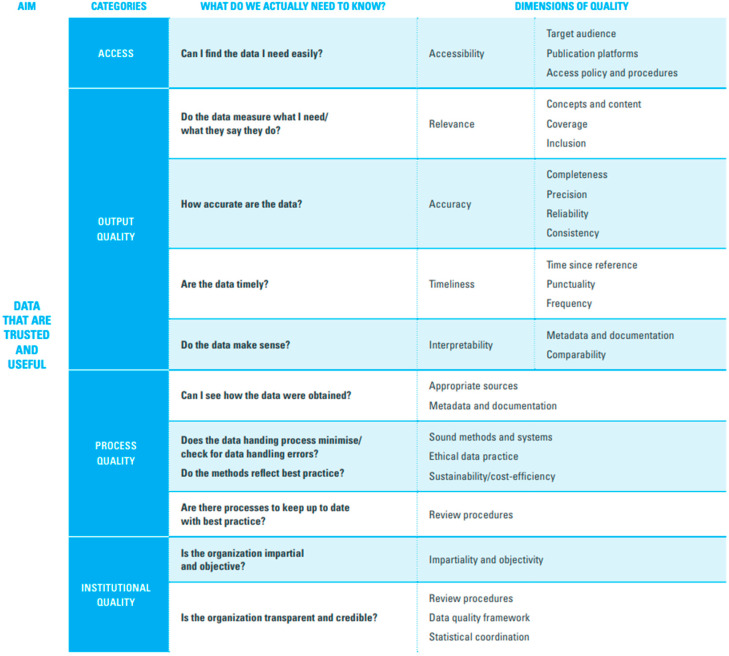
The UNICEF data quality framework includes access, output quality, process quality, and institutional quality. Adapted from the UNICEF data quality assessment framework.

We will compile a list of available data sources categorized by type, name, and the year in which the data were collected (Supplementary File, Template S1) [8]. Next, we will identify the available indicators associated with each dimension of inequality (Supplementary File, Template S2) [8]. From each data source, we will determine which dimensions of inequality are covered to assess data availability for monitoring these inequalities. Additionally, we will list the immunization indicators, with DTP3 coverage and zero-dose prevalence being the most used, included in each data source (Supplementary File, Template S3) [8]. If data sources do not specify that a quality assessment has been conducted, we will perform a data quality assessment using the UNICEF data quality framework (Figure 3) [15].

- Data linking

After identifying the data sources that contain indicators and dimensions of inequality, we will integrate all the information regarding immunization indicators and the associated dimensions of inequality to illustrate their connections. If either type of data is lacking, we will explore opportunities to link immunization indicators and dimensions of inequality from different sources. Linked data will be merged using individual or small area identifiers common to both sources. For example, different data sources containing personal identification numbers can be used to connect an individual’s immunization coverage with information about dimensions of inequality. Similarly, data sources with small-area identifiers, such as postal codes, will be linked to ecological data about the respective areas. If such identifiers exist, we will merge the sources accordingly.

#### 3.2.3. Data Analysis

Data analysis will involve two primary sub-steps: preparing disaggregated data and calculating summary measures of inequality [8].

- Disaggregated data preparation

We will utilize R Studio (Version 4.5.2) to clean the data by addressing outliers and missing values and removing any unnecessary parts of the data. Once the data is cleaned, we will define how to measure the immunization indicators, establish the number of subgroups for each dimension of inequality, and calculate disaggregated estimates that are broken down by population subgroups instead of presenting an overall average. When preparing the immunization disaggregated data, we will consider the specifications of the data sources, such as household surveys’ sampling designs (e.g., clustering, weighting, and stratification).

- Calculating summary measures of inequality

We will upload the disaggregated data to the WHO Health Equity Assessment Toolkit Plus (HEAT Plus) software (Version 7.0. Geneva: WHO, 2026.) to analyze immunization inequalities. We will use two primary measures of inequality: absolute measures (differences) and relative measures (ratios) for pairwise comparisons to capture various aspects of inequality in the immunization data.

#### 3.2.4. Reporting Results

We will visualize the inequality patterns in immunization data using graphs and tables tailored to our target audience [8]. Our results will be communicated in various formats specific to different audiences, including written reports, policy briefs, peer-reviewed publications, stakeholder engagement, and online interactive platforms (e.g., dashboards).

#### 3.2.5. Knowledge Translation

We will leverage inequality data to identify priority areas for action and propose strategies [8]. Our goal will be to promote the uptake of immunization inequality data among relevant policymakers, advocating for the integration of equity considerations into immunization policy processes.

## 4. Equity-Focused Health Policy Analysis Method

### 4.1. Policy Mapping and Analysis

This section aims to explain why inequalities persist despite the existence of programs; it does this by analyzing the structural factors that affect immunization outcomes, using the HPA triangle and the HEAP framework [6,16]. During the mapping exercise, we will assess a range of immunization policies and consider the following questions:

- What health policy or research topic is addressed in the literature?
- Is it person-centered or specific?
- Does it pertain to physical aspects (e.g., hospitals, clinics, health centers, birth centers)?
- Does it cover economic, sociocultural, or educational (e.g., health education) dimensions?
- Who or what groups are the focus of the policy?
- Individuals (e.g., pregnant women, mothers, adults, children, and neonates).
- Families.
- Subpopulations (e.g., ethnicity, communities, age groups).

By addressing these questions, we will be able to map and select eligible policies effectively.

After selecting relevant policies, we will use the HPA triangle to analyze the policy content, actors, policy processes, and the contextual factors that influence immunization outcomes. The HPA triangle will help us examine the content of immunization policies, the contextual factors that affect their impact, the decision-making process in immunization, and the actors involved in the policy processes. Contextual factors may include systemic and structural issues such as economic instability, conflict, and governance structures, all of which shape the immunization landscape.

Next, we will conduct stakeholder mapping and analysis to identify the power, position, and interests of various stakeholders in immunization topics. Following this, we will review the content of the immunization policies, focusing on their goals, objectives, target activities, and resource allocations. Recognizing that data transparency can be politically sensitive, this analysis goes beyond simple identification; it serves as a foundation for Problem-Driven Iterative Adaptation (PDIA). By focusing on locally nominated problems and seeking ‘small wins’ through iterative adjustments, the framework allows for progress even in environments where high-level policy change faces resistance. This ensures that the integration of HIM and HPA is not a rigid, top-down mandate, but a flexible process that builds a ‘coalition of the willing’ to champion data-driven immunization equity.

Additionally, we will utilize the HEAP framework along with inequality data from HEAT Plus to address questions related to how equity is incorporated into immunization policies.

### 4.2. Policy Intent and Framing

- What is the policy trying to do, and how does it conceptualize equity?
- What problem does the policy seek to address, and what are its stated objectives?
- How is equity defined within the policy?
- Does the policy explicitly aim to reduce disparities or inequities, or implicitly?
- Does it address social or economic determinants of health, and if so, which ones (e.g., access to care, education, housing, racism, transportation)?

### 4.3. Population and Geographic Impact

- Who is affected, and where?
- Which geographic areas are impacted (national, subnational, local)?
- Which populations live in those areas, particularly by race and ethnicity?
- Are some groups more likely to experience benefits or burdens from the policy?
- What data sources are used to identify affected populations?

### 4.4. Data Availability and Adequacy

- Is there sufficient evidence to assess equity impacts?
- Are the available data adequate to understand who will be affected?
- Are key data missing, insufficient, or poorly disaggregated?
- Could alternative or non-traditional data sources (e.g., community data, qualitative evidence) strengthen understanding?

### 4.5. Systemic Racism and Structural Inequities

- How does the policy interact with existing systems of disadvantage?
- Are inequities related to the policy issue disproportionately experienced by specific racial or ethnic groups?
- How might the policy promote racial equity?
- How might it unintentionally reinforce or exacerbate inequities?
- What changes could be made to better mitigate structural or systemic harms?
- In contexts where explicit data on race or ethnicity is unavailable or subject to political constraints, the framework addresses structural inequity by employing geographic and socio-economic proxies. Mapping immunization coverage alongside historical investment patterns and residential segregation, including ‘red-lining,’ enables the identification of spatial manifestations of systemic racism. Consequently, the HEAP framework maintains effectiveness by targeting structural drivers of exclusion, such as unequal access to transport, water, and sanitation, which frequently serve as quantifiable indicators of racial or ethnic disparities.

### 4.6. Community Engagement

- Were affected communities meaningfully involved?
- Were communities most affected by the policy engaged in its design?
- Was engagement representative and meaningful (i.e., able to influence decisions)?
- How was community input incorporated, or why was it not?
- Does the policy ensure ongoing engagement and communication over time?

### 4.7. Feasibility and Implementation

- Can the policy realistically deliver equitable outcomes?
- Have similar policies succeeded or failed in advancing equity elsewhere?
- Does the policy account for staffing, budget, and resource requirements?
- Are the likely implementation barriers identified and addressed?
- What additional resources or actions are needed to ensure equity goals are met?

### 4.8. Accountability and Governance

- Who is responsible for equity outcomes, and how are they monitored?
- Does the evaluation plan include assessment of equity impacts?
- Are there mechanisms to assess impacts on specific communities over time?
- Is there a designated individual or unit responsible for centering equity?
- Are decision-makers accountable to both institutions and affected communities?
- How, how often, and with whom will results be shared?

### 4.9. Overall Equity Assessment and Recommendations

- Should this policy move forward from an equity perspective?
- Which groups are more likely to benefit or be burdened?
- What evidence supports these conclusions?
- Should the policy proceed as-is, be modified, or not be pursued?
- What concrete recommendations would improve its equity impact?

## 5. Informatics-Enabled Data-to-Policy Pathway

**Figure 4:**
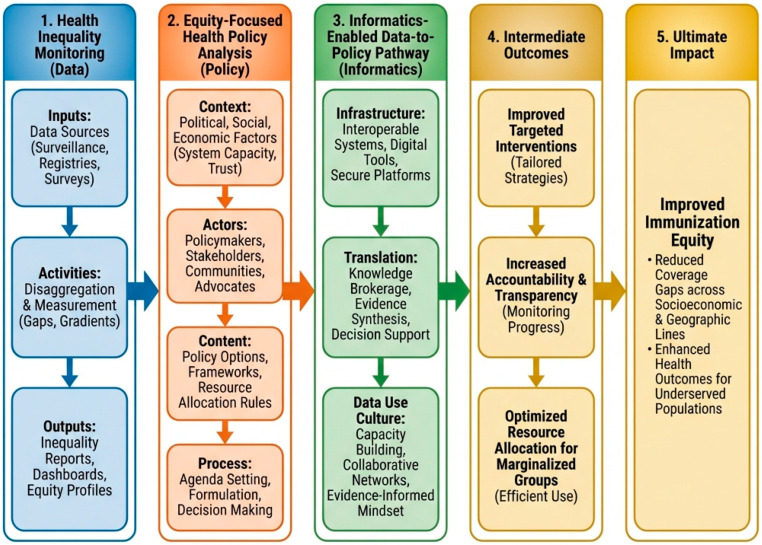
Integrated informatics-enabled data-to-policy framework for immunization equity. Pathways of Change shown by the arrows illustrate how Health Inequality Monitoring (HIM) feeds into Equity-Focused Health Policy Analysis, which in turn feeds into the Informatics-Enabled Data-to-Policy (D2P) pathway. *This figure was created using Canva AI—Magic Studio. https://www.canva.com/*.

This subsection outlines the methodology for integrating monitoring data and policy analysis to facilitate actionable change. It is based on the Integrated informatics-enabled data-to-policy framework for immunization equity (Figure 4). The informatics foundation encompasses infrastructure, such as using platforms like DHIS2 to collect real-time, detailed facility data, allowing quick detection of coverage gaps [16]. It also includes interoperability, which ensures that data systems can communicate with each other to provide a complete picture of health factors [17]. The translation layer (knowledge to action) includes knowledge creation, which synthesizes disaggregated data and policy analysis into policy briefs and recommendations [11]. It also involves economic evaluation, including root-cause analysis and cost-effectiveness modelling to present financially viable equity interventions to decision-makers [18]. Finally, data use culture (behavioural change) entails capacity building as a way of institutionalizing data practices, transforming ad hoc data use into a consistent culture where data is regularly needed for planning and budgeting [11,19]. Capacity building includes training health staff to interpret visualizations and empowering them to act on the information [18]. Additionally, feedback loops can be established through mechanisms that ensure data circulates back to the facility or district level, informing local microplanning efforts [4].

This conceptual framework combines the precision of inequality monitoring with the detailed insights of policy analysis, connected by an informatics-driven approach that transforms data into actionable outcomes. Figure 4 illustrates the integrated conceptual framework supporting this protocol, demonstrating the journey from monitoring health inequalities to analyzing equity-focused policies, culminating in an informatics-enabled data-to-policy approach that turns data into actionable outcomes. The framework conceptually builds on established WHO methods for monitoring health inequalities and analyzing equity-focused policies [8].

## 6. Outputs and Deliverables

### 6.1. Analytical Outputs: The “State of Inequality” Evidence Base

The primary analytical output will be a comprehensive health inequality assessment report. This report will provide essential evidence, highlighting the extent and distribution of immunization disparities.

- Disaggregated immunization estimates: datasets and visual summaries that show coverage rates broken down by key dimensions of inequality, such as economic status, education, subnational region, and gender.
- Summary measures of inequality: technical annex reporting calculated metrics for simple (absolute and relative measures) and complex measures of inequality (e.g., slope index of inequality and relative index of inequality), to quantify the gradients of disadvantage across the population.
- Root cause and contextual analysis: a qualitative report that uses the HPA and HEAP frameworks to identify the policy content, actors, context, and processes impacting immunization outcomes. It also features a stakeholder analysis matrix that highlights the power relationships and positions of key players regarding equity efforts.

### 6.2. Research Translational Products: Policy Briefs and Economic Evaluations

To facilitate knowledge translation (KT), the project will produce concise, decision-focused outputs designed for non-technical policymakers, adhering to the D2P curriculum standards.

- Evidence-based policy briefs: these one- to three-page documents identify the inequality issue using local data, compare policy options, and suggest clear, actionable steps. They aim to explain the scope of the problem, highlight changeable root causes, and offer practical solutions.
- Economic evaluation models: these include cost-effectiveness analyses, such as incremental cost-effectiveness ratios, which are used as decision-making aids. They assess the financial costs and health benefits, e.g., the cost per death prevented, of proposed equity strategies to support resource prioritization.
- Health impact assessment: a forward-looking evaluation that estimates how proposed policies might affect vulnerable groups, ensuring the incorporation of the “do no harm” principle into policy development.

### 6.3. Informatics Tools: Dashboards and Visualizations

To facilitate ongoing monitoring and operational decision-making, the project will provide configured informatics tools that can be integrated into the national health information system infrastructure, such as DHIS2.

- Equity dashboards: interactive, web-based visualization tools that enable users to query multiple datasets and filter results by equity stratifiers, such as mapping zero-dose children according to district poverty levels. These dashboards employ “traffic light” or scorecard visualizations to highlight underperforming districts, prompting immediate management actions.
- Integrated data repository: a centralized, interoperable data warehouse that connects facility-level service data with population-level surveillance and survey data. It includes essential metadata dictionaries to maintain data traceability and ensure quality.

## 7. Dissemination and Capacity-Building Mechanisms

To ensure the sustainability and institutionalization of the framework, the project will provide structured engagement and learning outputs.

- Policy dialogues and forums: organized events where researchers and policymakers discuss policy brief findings to align research questions with political priorities and create opportunities for policy change.
- Training curriculum and materials: a localized “Training of Trainers” program based on the D2P methodology, designed to train health personnel in root cause analysis, health impact assessment, economic evaluation, and other relevant skills.

## 8. Strengths and Limitations of the Protocol

The proposed concept utilizes a multimethod approach that combines extensive quantitative monitoring with qualitative policy analysis to create a comprehensive and action-oriented design. One of its main advantages is the ability to go beyond mere descriptions and facilitate actionable policy changes. A key strength of the approach is its use of the Data-to-Policy (D2P) theory of change, which directly links technical data analysis to the production of decision-oriented outputs, such as policy briefs. This addresses the common issue of health data being underutilized or unprocessed by decision-makers.

By integrating the WHO HIM framework with policy analysis tools, including the HPA and HEAP frameworks, the protocol assesses inequality while also exploring its underlying causes. This approach considers the policy content, context, actors, and processes, providing a holistic understanding of the root causes of immunization inequities.

The concept employs a robust quantitative methodology, utilizing simple (absolute and ratio) and complex summary measures like the slope index of inequality and the relative index of inequality. The complex measures help capture the full spectrum of inequality across the entire population, while simple measures compare extreme groups, such as the wealthy versus the poor. This methodology also adopts an intersectional focus to address a critical gap in standard monitoring, allowing for the identification of subgroups facing multiple layers of disadvantage, which are often obscured by national averages.

To enhance technological feasibility and scalability, the concept will leverage existing infrastructure, specifically using DHIS2 as the informatics backbone. This is a significant advantage because DHIS2 is an open-source, scalable platform that is widely adopted in resource-constrained settings. It enables real-time data collection and reduces the need for costly new software development. Additionally, the approach maximizes interoperability by incorporating data exchange standards, supporting the establishment of an integrated data repository that facilitates the triangulation of facility data with population surveys, crucial for validating immunization coverage.

Furthermore, the concept promotes sustainability by possibly developing the ministries of health’s workforce through embedded training. This “training of trainers” method establishes a group of local experts skilled in ongoing root cause analysis and economic evaluation, thereby reducing dependence on outside consultants.

Despite its robust design, the concept faces significant challenges related to data quality and resource constraints. A major limitation of using routine health information systems like DHIS2 is the frequent exclusion of data from private providers, which creates a “blind spot” in coverage assessments, particularly in urban regions where private healthcare use is common. The concept relies on secondary data that may overstate vaccine coverage, especially in systems where funding is linked to performance results.

To address the ‘urban blind spot’ in administrative datasets like DHIS2, the framework uses triangulation. In areas with high private-sector health use, such as the Western Cape’s urban hubs, administrative coverage will be cross-referenced with household survey data and private-sector vaccine procurement records. This helps estimate ‘hidden’ coverage. The framework also supports public–private data exchange, calling for private-sector vaccinations to be included in the national Electronic Immunization Registry. This ensures ‘zero-dose’ status reflects a child’s actual lack of protection, not just their absence from public-sector reporting.

Monitoring inequality can be politically sensitive; political actors might suppress or overlook data that reveal significant disparities or service failures to avoid criticism or damage to public trust. Additionally, the HPA triangle may indicate that the responsibility for immunization and equity is dispersed across various government departments, such as health, finance, and social welfare, complicating coordination and hindering the implementation of policy recommendations. Finally, the sustainability of the informatics pathway is jeopardized by high staff turnover in the IT and health sectors, leading to a loss of institutional knowledge and the need for ongoing costly retraining.

## 9. Conclusions

Persistent immunization inequalities necessitate stronger linkages between evidence generation and policy action. Although HIM has advanced the systematic measurement of disparities, its impact is often diluted by poor integration into HPA processes. This conceptual framework addresses this gap by proposing a structured, informatics-enabled approach to translate inequality data into equity-oriented policies and programs.

By synthesizing data production, stakeholder engagement, and iterative review, the framework emphasizes the necessity of interconnected information systems in promoting evidence use. It redefines inequality data, shifting it from a descriptive output to an actionable input for resource allocation and accountability. Adopting this integrated model supports countries, particularly LMIC settings, in operationalizing the equity commitments of Immunization Agenda 2030 (IA2030). Future research must focus on empirically validating this framework across diverse contexts and research fields, embedding equity-focused evidence within routine decision-making. Although this framework provides a comprehensive structure for integrating health information and policy analysis, its effectiveness is best evaluated through practical application. To advance beyond theoretical development, we are implementing this approach in the Western Cape Province of South Africa. By integrating household survey data with administrative records, we seek to identify the specific dimensions of inequality that contribute to the prevalence of ‘zero-dose’ children. This pilot aims to translate informatics into actionable, localized insights, equipping health leaders with the resources necessary to design more equitable and targeted immunization programs for the most underserved children.

In the Western Cape, this coordination will use established inter-governmental structures. These include the Provincial Health Council. The goal is to ensure that HIM data on zero-dose children informs clinical outreach and interdepartmental social support programs. Existing infrastructure needs are mostly met by the Provincial Health Data Centre, in the Western Cape. The main investment needed is in people. Specifically, data analysts need training to do more than just aggregate reporting. They must also be able to conduct the equity-focused policy analysis described in this framework.

## Data Availability

The original contributions presented in this study are included in the article/Supplementary Materials. Further inquiries can be directed to the corresponding authors.

## Supplementary Materials

The supporting information can be downloaded at: https://www.mdpi.com/article/10.3390/vaccines14030219/s1, Template S1: Data sources list; Template S2: Inequality dimension; Template S3: Health indicators.
